# Association between dentition defects and Alzheimer’s disease risk: a systematic review and meta-analysis

**DOI:** 10.3389/fdmed.2026.1783171

**Published:** 2026-04-20

**Authors:** Qianxia Wang, Kaiyan Zhou, Miaomiao Zhang, Yao Dong, Mei Zhao

**Affiliations:** 1 College & Hospital of Stomatology, Anhui Medical University, Hefei, Anhui Province, China; 2 Anhui Provincial Key Laboratory of Oral Diseases Research, Hefei, Anhui Province, China

**Keywords:** Alzheimer's disease, dentition defects, meta-analysis, systematic review, tooth loss

## Abstract

**Background:**

Alzheimer's Disease (AD) is the most common neurodegenerative disorder among the elderly, with a steadily rising prevalence that poses a significant global public health challenge. Recently, dentition defects (DD), such as tooth loss, have gained attention as potential risk factors influencing neurocognitive health. However, the relationship between DD and AD remains inconclusive, necessitating a systematic analysis to clarify this association.

**Methods:**

This systematic review and meta-analysis was conducted in accordance with PRISMA guidelines. We searched PubMed, Embase, Web of Science, and Cochrane Library databases to identify relevant studies published between 1996 and 2022. Eligible studies assessing the relationship between DD and AD were included. A random-effects model was applied to estimate the pooled odds ratios (ORs) with 95% confidence intervals (CIs). Heterogeneity and publication bias were also assessed.

**Results:**

Fourteen studies were included, with sample sizes ranging from 52 to 156,450 participants. The meta-analysis revealed a significant association between DD and an increased risk of AD (OR=1.38, 95% CI: 1.09–1.74, *P* < 0.05). Heterogeneity among the studies was substantial (I² = 97%), reflecting considerable variability in study designs, populations, and exposure definitions. Sensitivity analysis and publication bias assessments indicated the reliability of the results despite high heterogeneity. Mechanistic analyses suggested that DD may elevate AD risk through pathways such as chronic inflammation, nutritional deficiencies, alterations in the oral microbiome, and reduced cognitive reserve.

**Conclusions:**

DD are significantly associated with an increased risk of AD. Improving oral health may represent a modifiable factor warranting. However, further high-quality prospective studies are needed to validate these findings and explore the underlying mechanisms.

## Introduction

Alzheimer's disease (AD) is a progressive neurodegenerative disorder that represents one of the most pressing public health challenges of the 21st century. As the leading cause of dementia among older adults, AD currently affects more than 55 million people worldwide. With the rapid aging of the global population, its prevalence is projected to double every 20 years, reaching an estimated 131.5 million by 2050 (1). The disease is characterized by progressive memory loss, cognitive decline, and behavioral changes, which irreversibly deprive individuals of their cognitive autonomy and personal identity. Beyond its profound impact on patients, AD imposes a heavy emotional and financial burden on families and societies. The total global societal cost of dementia is estimated to exceed US$1 trillion annually, underscoring its profound socioeconomic implications (2).

Among the numerous known risk factors associated with AD, dentition defects (DD), particularly multiple-tooth loss and edentulism, have attracted increasing research attention in recent years. Epidemiological observations suggest that oral health status and cognitive function often decline concurrently during the natural aging process, indicating not only a correlation but also a potential bidirectional and interactive relationship (3). The oral cavity is a critical microbial habitat whose health status is closely linked to a wide range of systemic conditions. It is noteworthy that DD is highly prevalent among older adults, with approximately 30% of individuals aged 65 and above worldwide suffering from severe periodontitis accompanied by significant tooth loss (4). This demographic substantially overlaps with the population at high risk for AD, further emphasizing the public health relevance and urgency of investigating the potential link between oral health and cognitive decline.

The consequences of DD extend far beyond local impairments in mastication, speech articulation, and facial aesthetics. Chronic oral infections resulting from poor oral health, such as periodontitis, can induce a persistent state of low-grade systemic inflammation, reflected in elevated serum levels of inflammatory markers such as C-reactive protein and interleukin-6 (1). This chronic systemic inflammation is widely regarded as a key mechanistic bridge linking peripheral diseases to central nervous system pathology. Studies have demonstrated that systemic inflammation can exacerbate or directly trigger neuropathological processes characteristic of AD through multiple pathways, including disruption of the blood-brain barrier integrity, microglial activation, and promotion of β-amyloid deposition and Tau hyperphosphorylation (5). Furthermore, oral pathogens such as Porphyromonas gingivalisand their virulence factors have been detected in the brain and are hypothesized to act as potential “pathogenic triggers” in AD pathogenesis (6).

The 2020 Lancet Commission identified 12 potentially modifiable risk factors—including less education, hypertension, hearing impairment, smoking, obesity, depression, physical inactivity, diabetes, low social contact, excessive alcohol consumption, traumatic brain injury, and air pollution—that together are associated with approximately 40% of dementia cases worldwide (2). Although increasing attention has been paid to the relationship between oral health and systemic conditions, dentition defects (DD) have not yet been incorporated into this framework. This omission is noteworthy given that DD shares several biological and behavioral correlates with established risk factors, including chronic inflammation (similar to mechanisms implicated in hypertension and diabetes), nutritional compromise (overlapping with metabolic disorders), and reduced social engagement (paralleling low social contact) (7). Considering DD within this broader risk context may enrich understanding of dementia-related vulnerability by highlighting oral health as part of the complex network of factors associated with cognitive decline. Rather than implying a direct preventive effect, this perspective underscores the interconnectedness of oral and systemic health within aging populations. Evidence from Mendelian randomization studies has also suggested a potential association between occlusal dysfunction and Alzheimer's disease, although such findings should be interpreted cautiously and do not establish definitive causality (8). Overall, these observations support further investigation into the role of oral health in dementia risk trajectories, particularly within longitudinal and mechanistic research frameworks.

Despite these plausible pathophysiological connections, existing evidence remains inconsistent. Several large-scale prospective cohort studies and meta-analyses have failed to identify a statistically significant and independent association between tooth loss and increased risk of AD (9). These discrepancies suggest that the relationship may be confounded by factors such as educational attainment, socioeconomic status, comorbidities, and lifestyle behaviors, or may be evident only in specific subpopulations. Such controversy highlights methodological heterogeneity across studies in terms of design, population definition, and measurement of exposures and outcomes. It also underscores the need for more rigorously designed longitudinal studies to clarify causal relationships and elucidate potential mediating and moderating mechanisms.

In conclusion, the relationship between DD and AD is complex, multifactorial, and not yet fully understood. Although compelling inflammatory and microbial pathways have been proposed, conclusive epidemiological evidence remains incomplete. To integrate currently conflicting findings and provide a clearer direction for future mechanistic investigations and public health interventions, a systematic synthesis of the available observational evidence is both timely and necessary. Therefore, this study aims to conduct a systematic review and meta-analysis to comprehensively identify, critically appraise, and quantitatively synthesize epidemiological evidence on the association between DD—particularly tooth loss—and the risk of developing Alzheimer's disease, thereby providing high-level scientific evidence to inform integrated prevention strategies that incorporate oral health into cognitive aging initiatives.

## Methods

### Protocol registration

This investigation has been duly registered with PROSPERO, the International Registry of Systematic Reviews, under the registration number CRD42022333663 (available at: https://www.crd.york.ac.uk/PROSPERO/). We have meticulously conducted this meta-analysis adhering to the PRISMA 2021 guidelines, which set the standard for systematic evaluations and meta-analyses (Supplementary Table S1).

### Search strategy

We conducted a comprehensive search of PubMed, Embase, Web of Science, and the Cochrane Library databases, which extended until March 1, 2026, without any language exclusions. Our search strategy utilized both MeSH terms and free-text keywords to encompass the breadth of literature on AD and related dementia subtypes. The search terms included: “Alzheimer Dementia” [MeSH], “Alzheimer Dementias”, “Dementia, Alzheimer”, “Alzheimer's Disease”, “Dementia, Senile”, “Senile Dementia”, “Dementia, Alzheimer Type”, “Alzheimer Type Dementia”, “Alzheimer-Type Dementia (ATD)”, “Alzheimer Type Dementia (ATD)”, “Dementia, Alzheimer-Type (ATD)”, and “Alzheimer Type Senile Dementia”, combined with “dentition defects” [MeSH]. A detailed account of our search methodology is presented in Supplementary Table S2.

### Study selection

Following the database search, we imported the retrieved studies into Endnote X9 software (Thomson Reuters, New York, NY, USA) for further organization and management. The preliminary screening of titles and abstracts was conducted by an initial reviewer (Q.X-W) and subsequent verification was carried out by a second reviewer (M.M-Z), ensuring reliability in study selection. Prior to the commencement of data extraction and quality assessment, full texts were rigorously appraised. Both authors conferred on the selection of studies for inclusion, and any discrepancies were reconciled through comprehensive discussions. In preparation for the database search, we meticulously designed a data extraction template to capture essential details from each study, encompassing demographic data, data sources, exclusionary specifications, durations of follow-up, diagnostic benchmarks, and metrics of outcomes.

Rigorous inclusion criteria were established to direct the search, which aimed at consolidating pertinent studies, curtailing heterogeneity, and enhancing the robustness of the findings. The selection parameters were as follows: (1) studies involving adult patients presenting with DD, inclusive of tooth loss (DD include multiple dimensions, such as tooth loss, insufficient occlusal support, and impaired masticatory function); (2) studies with a methodological framework that encompassed cross-sectional analyses, retrospective or prospective cohort studies, and randomized controlled trials.

In line with the rigorous PICOS framework, the selection criteria for studies examining the connection between DD and AD were meticulously defined as follows: Participants: (1) The investigation was confined to adult participants. (2) Exposure and Comparison: The observational cohort comprised individuals exhibiting dental anomalies, inclusive of tooth loss, contrasted against counterparts devoid of such conditions. (3) Outcomes: The key endpoint assessed was the potential association between DD and the incidence of AD. (4) Study Designs: retrospective and prospective cohort studies, case-control assessments, and randomized controlled trials for inclusion. Conversely, studies were omitted if they fell under any of the subsequent categories: (1) Descriptive articles such as protocols, narrative reviews, conference abstracts, or research of veterinary focus. (2) Investigations that failed to provide sufficient data, where further requisite details remained inaccessible after communication attempts with the study's corresponding authors.

### Data extraction and quality assessment

The extraction of data was meticulously undertaken by two researchers (Q.X-W and K.Y-Z), who independently gathered essential information from each selected study. The details collated included the following: (1) First Author; (2) Year of Publication; (3) country. (4) Methodology; (5) Duration of Observation; (6) Population Demographics; (7) Criteria for Dental Deficiency Diagnosis; (8) outcomes; (9) Confounding Adjustments; (10) Relative Risk (RR), Hazard Ratio (HR), or Odds Ratio (OR) accompanied by the 95% Confidence Intervals (CIs) in the context of the adjusted analysis.

To assess the methodological integrity and quality of the cohort studies, we adopted the Newcastle-Ottawa Scale (NOS). Studies that scored above 6 on this scale were considered to be of satisfactory quality. Two of our researchers (M.M-Z and D.Y) carefully evaluated the quality of evidence for individual outcomes.

### Statistical analysis

Due to anticipated clinical and methodological diversity across included studies—including variations in study design (case-control, retrospective cohort, prospective cohort), populations (different countries and healthcare settings), definitions of dentition defects (tooth loss, occlusal support, masticatory function), and adjustment for confounders—we employed a random-effects model (DerSimonian and Laird method) for all analyses. The random-effects model assumes that the true effect sizes vary across studies according to a distribution, rather than sharing a common fixed effect, and provides a more conservative estimate that accounts for both within-study and between-study variance25. This approach is particularly appropriate when studies are drawn from populations that differ in ways that could influence the true effect size, as is the case with the heterogeneous observational evidence on DD and AD.

We equated Relative Risks (RRs) and Hazard Ratios (HRs) with Odds Ratios (ORs), aggregating the summary ORs along with their 95% Confidence Intervals (CIs) through the inverse-variance approach. We combined ORs, RRs, and HRs in the meta-analysis under the assumption that AD is a relatively rare outcome in the general population (incidence < 10%), such that ORs approximate RRs and HRs (10). For cohort studies reporting HRs, we considered these to be equivalent to RRs and used them directly in the meta-analysis. For case-control studies, we used reported ORs. We did not apply additional corrections for study design, as the primary analysis aimed to estimate the direction and magnitude of association rather than causal effects. However, we acknowledge that combining different effect measures may introduce bias due to differential adjustment for confounders and varying susceptibility to design-specific biases (e.g., recall bias in case-control studies, loss to follow-up in cohort studies). Heterogeneity was evaluated using the Higgins I-squared (*I*^2^) statistic (30, 50, and 75% represent low, moderate, and high heterogeneity, respectively). Publication bias was addressed by the funnel plot, and Egger's and Begg's tests. To appraise the robustness and reliability of the primary study outcomes, we also carried out sensitivity analyses by omitting each study in turn. Subgroup analysis was defined as type of research (cohort study and case-control study) and region (Asia, Europe, North America and South America).

The statistical analysis was performed by RevMan software, version 5.4.1 (The Cochrane Collaboration, Nordic Cochrane Center,Copenhagen, Denmark) and Stata software, Version 16.0 (Stata Corp. LP, College Station, TX, USA). *P* < 0.05 double-sided was considered statistically significant.

### GRADE assessment

We assessed the certainty of evidence for the primary outcome using the Grading of Recommendations Assessment, Development and Evaluation (GRADE) framework (11). Evidence was evaluated across five domains: risk of bias, inconsistency, indirectness, imprecision, and publication bias. Certainty was rated as high, moderate, low, or very low according to standard GRADE methodology. Two author separately assessed the quality of evidence profile tables. Evidence profile tables were generated using GRADEpro GDT, in which the results of the outcomes are presented as described in the outcome metric type section, and footnotes are used to indicate reasons for degradation or escalation of the quality of evidence.

## Results

### Literature search

**Figure 1:**
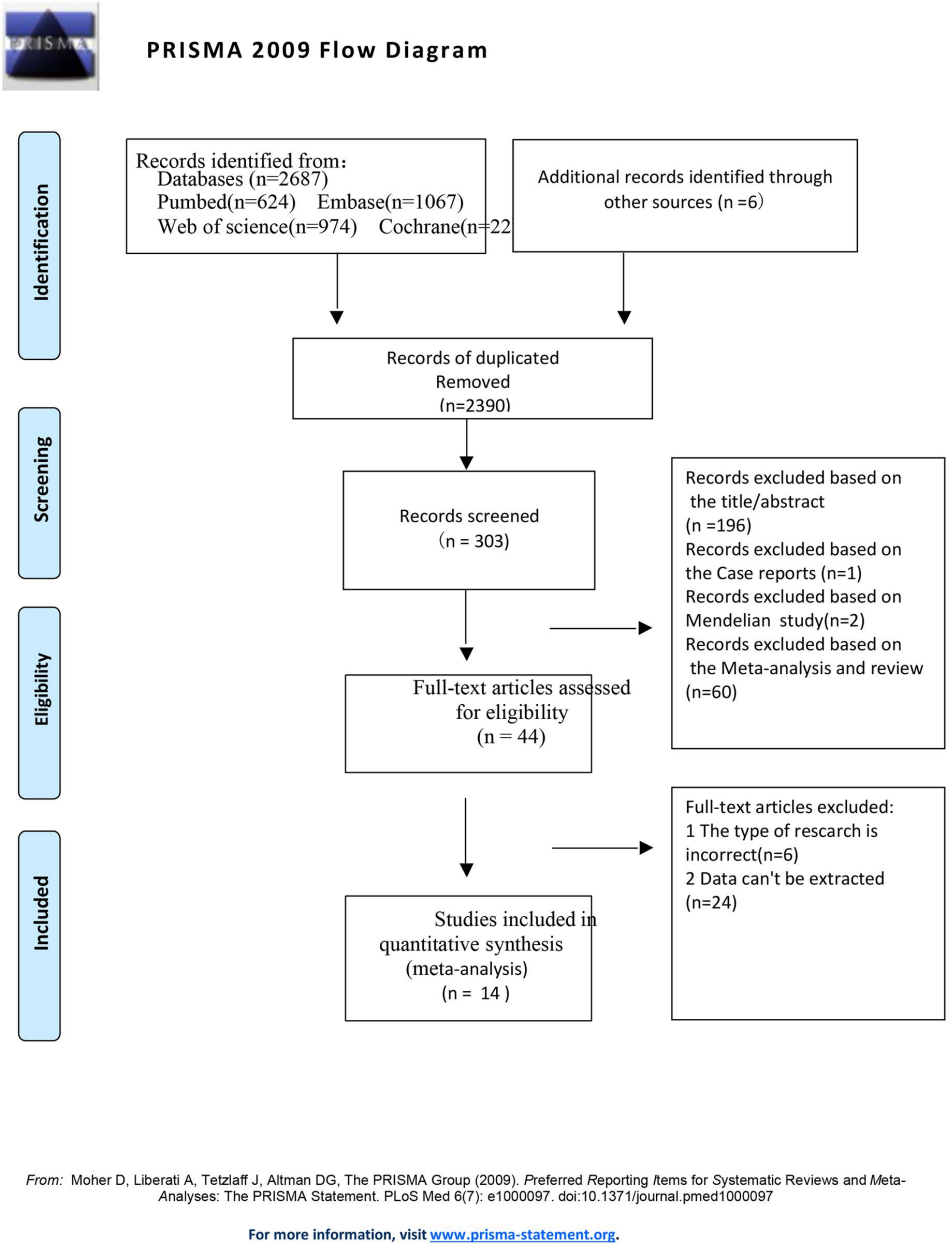
Flowchart of the study selection for the meta-analysis of an association between Alzheimer's disease and dentition defects.

Figure 1 illustrates our search and selection process for the relevant literature. An initial query of electronic databases yielded a total of 2,693 articles. Subsequent to removing 2,390 duplicates and 259 studies that did not meet the inclusion criteria, we were left with 44 articles for comprehensive full-text evaluation. Out of these, 30 studies were excluded based on the following reasons: (1) Date can’t be extracted (*n* = 24); (2) The type of research is inappropriate (*n* = 6). These exclusions, along with their respective rationales, are systematically documented in Supplementary Table S3. After this rigorous screening process, 14 (12–25) studies ultimately qualified for inclusion in our meta-analysis.

### Study characteristics and quality

Table 1 summarizes the main characteristics of the included studies. The studies included were published between 1996 and 2022, with sample sizes ranging from 52 to 156,450 participants. Most studies were conducted in Europe (*n* = 7), followed by Asia (*n* = 4), North America (*n* = 2), and South America (*n* = 1). Regarding study design, 10 were case-control studies, 3 were retrospective cohort studies, and 1 was a prospective cohort study. The follow-up periods varied from 1 to 15 years, though some studies did not report a follow-up period. The mean age varied from 50 to 87 years. Across all included studies, tooth loss was diagnosed primarily through clinical examination, and outcomes such as AD or cognitive impairment were defined based on clinical diagnosis or cognitive records. Among the studies reporting DD and AD, two studies reported crude risk estimates, and the other adjustments varied considerably. Six studies adjusted for significant confounding factors, including age, sex, income, region, educational level, tobacco and alcohol consumption, and 6 studies adjusted for confounders associated with AD, including hyperlipidemia, diabetes, hypertension, high cholesterol, clinical depression, CAD, CHF, severity of dementia, ischemic cardiopathy and stroke.

**Table 1:** Characteristics of included studies in this meta-analysis.

Author, year, country	Study design	Total population	Case (N)	Age (mean years)	Exposure	Tooth Loss diagnosis	Outcome	Total study follow-up period (years)	OR/RR/HR (95% CI)	Adjusting factors
Chun Hung Chu et al., 2015, Hong Kong	Case control study	118	59	80	Missing teeth	Clinical examination	AD	NA	1.08 (0.95–1.23)	Age, gender
					Edentulism				1.30 (0.47–3.57)	
Ji Hee Kim et al., (21), Korea	Case control study	39,810	7,962	≥60	Missing teeth	Clinical examination	AD	NA	1.15 (1.07–1.23)	Age and sex, income and region of residence.
Gil-Montoya et al., (18), Spain	Case control study	409	180	>50	Missing teeth	Clinical examination	AD	2	1.30 (1.21–1.39)	Age, sex, education level, oral hygiene habits, and the presence of hyperlipidemia
Nordcnrani et al., 1996, Sweden	Case control Study	80	40	87	Missing teeth	Clinical examination	AD	NA	1.08 (0.91–1.27)	Age, gender
					Edentulism				1.25 (0.37–4.20)	
F. Aragón et al., (12), spain	Case control study	106	70	AD:77.4 control:62.6	Missing teeth	Clinical examination	AD	1.3	5.45 (4.65–6.38)	Age
Warren et al., (24), USA	Case control study	133	45	80.9	Missing teeth	Clinical examination	AD	1.9	1.51 (1.33–1.71)	——
					Edentulism				5.74 (2.68–12.27)	
Sung Eun Choi et al., (15), USA	Retrospective cohort study	156,450	9,456	72 ± 6.9	Edentulism	Clinical records	cognitive impairment	5	1.26 (1.09–1.45)	Age at the beginning of the study period, diabetes, hypertension, high cholesterol, clinical depression, and history of smoking
Balwant Rai et al., (14), North Indian	Case control study	52	20	58–69	Edentulism	Clinical examination	AD	NA	0.48 (0.13–1.78)	Education, age, occupation, BMI, CAD, CHF and diabetes,
Souza Rolim et al., (23), Brazil	Case Control Study	59	29	AD:61.17 control:75.17	Edentulism	Clinical examination	AD	1	0.69 (0.24–1.97)	Age, gender
Gil-Montoya et al., (19), Spain	Case control study	564	240	AD:78.25 control:79.8	Edentulism	Clinical examination	AD	NA	2.88 (1.97–4.22)	Age, sex, educational level, and tobacco and alcohol consumption
Syrjäla et al., (17), Finland	Case control study	354	49	82	Edentulism	Clinical examination	AD	NA	1.0 (0.4–2.5)	Adjusted for age, gender, education, smoking, severity of dementia and type of dwelling.
Arriveé et al., (13), French	Retrospective cohort study	405	72	66–80	Missing teeth	Clinical examination	AD	15	1.07 (0.57–2.02)	Gender, body mass index, diabetes, depression, hypertension and ischemic cardiopathy⁄history of brain stroke
Andreas Zenthöfer et al., (25), Germany	Case control study	90	33	AD:81.7 control: 83.4	Missing teeth	Clinical examination	AD	NA	1.01 (0.86–1.18)	——
Kim et al., (20), Korea	Prospective cohort study	686	57	≥65	Missing teeth	Clinical examination	AD	2.4	1.26 (1.00–1.59)	Age, gender and education, reported diet, vascular disease/risk, BMI and mid-arm circumference, albumin and cholesterol

AD, Alzheimer's disease; CAD, coronary artery disease; CHF, congestive heart failure; BMI, body mass index; OR, odds ratio; RR, relative risk; HR, hazard ratio; NA, not application.

The quality of the included studies was assessed using the Newcastle-Ottawa Scale (NOS), with total scores ranging from 5 to 8, indicating moderate to high methodological quality overall (Supplementary Figure S4). In the selection domain, most studies demonstrated good representativeness of the study population and appropriate group selection; however, some studies lacked detailed descriptions of how DD were assessed. In the comparability domain, most studies adequately controlled for age and at least one other confounding factor, though a few studies did not fully account for all potential confounders. In the outcome domain, while most studies had sufficient follow-up durations, a few were limited by inadequate follow-up.

### Alzheimer’s disease and dentition defects

**Figure 2:**
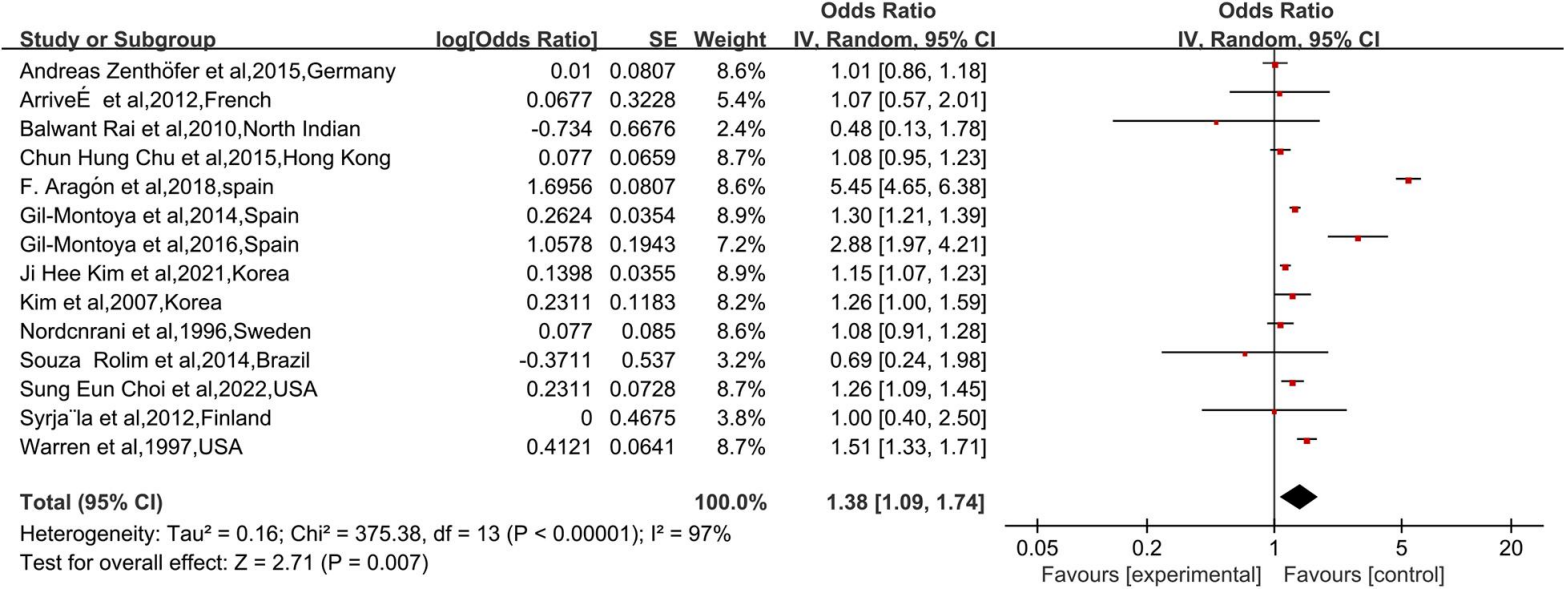
Forest plot of the risk of AD in patients with DD. In the forest plot, the diamond indicates the pooled estimate. Red boxes are relative to study size and the black vertical lines indicate 95% CIs around the effect size estimate.

A total of 14 studies reported the risk of new-onset Alzheimer's disease among dentition defects patients. As show in Figure 2, the risk of Alzheimer's disease was significantly increased in dentition defects patients compared with non-dentition defects patients in pooled studies (OR: 1.38, 95% CI: 1.09–1.74, *I^2^* = 97%), with substantial heterogeneity (*I^2^* = 97%, *τ*² = 0.16, *P* < 0.001). The 95% prediction interval for the overall effect ranged from 0.61 to 3.12, indicating that the true effect in a future study could lie outside the confidence interval and underscoring the need for cautious interpretation.

**Figure 3:**
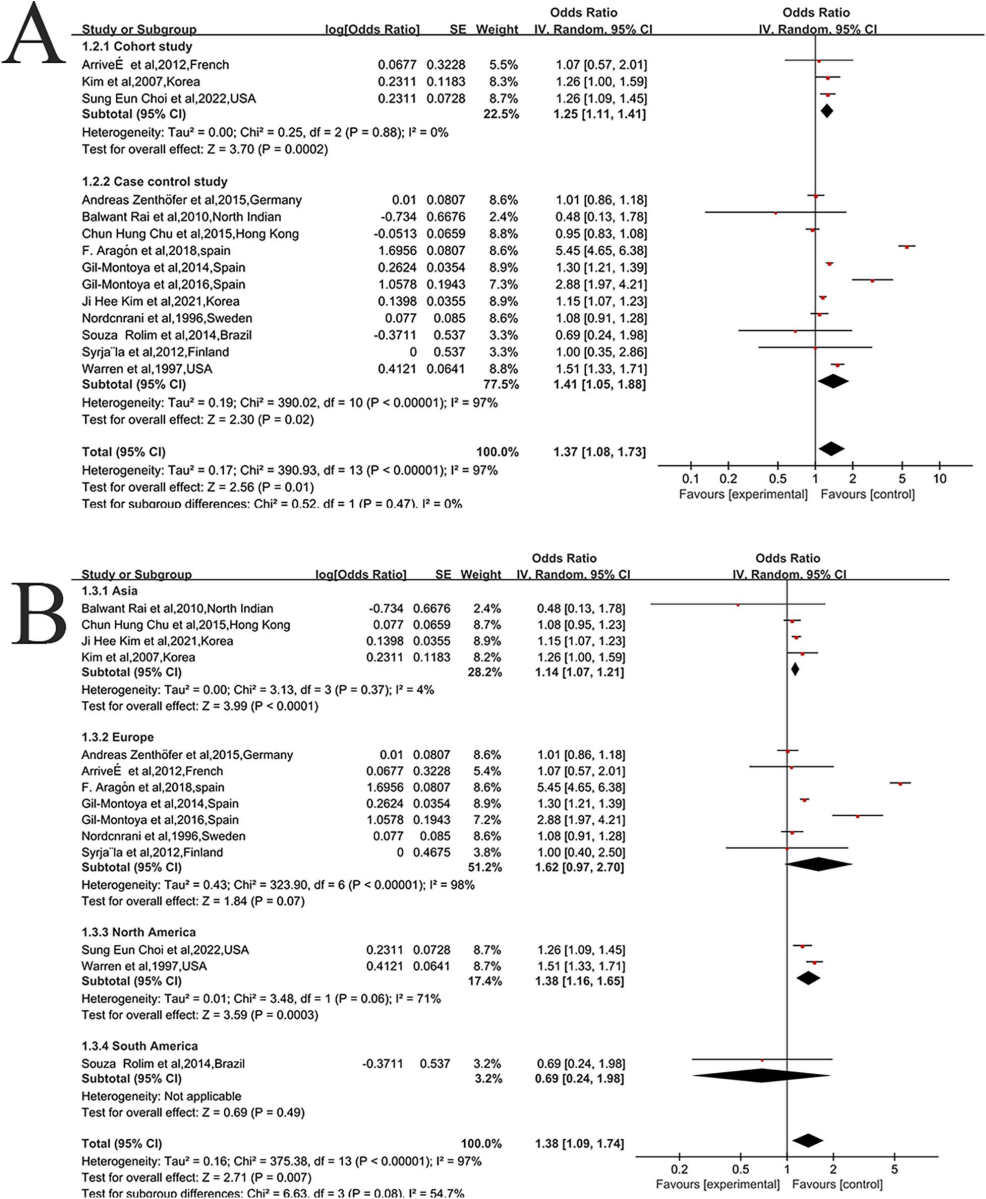
Forest plot of subgroup analysis of Alzheimer's disease in patients with dentition defects [**(A)** study design, and **(B)** region]. In the forest plot, the diamond indicates the pooled estimate. Red boxes are relative to study size and the black vertical lines indicate 95% CIs around the effect size estimate.

Subgroup analyses were performed according to study design, geographic region. Specifically, the pooled OR was 1.25 (95% CI: 1.11–1.41, *I^2^* = 0%) in cohort studies and 1.41 (95% CI: 1.05–1.88, *I^2^* = 97%) in case–control studies (Figure 3A). The association was also observed in both Asian (OR: 1.14, 95% CI: 1.07–1.21, *I^2^* = 4%) and North America (OR: 1.38, 95% CI: 1.16–1.65, *I^2^* = 71%), but not in studies from Europe (OR: 1.62, 95% CI: 0.97–2.70, *I^2^* = 98%), South America (OR: 0.69, 95% CI: 0.24–1.98, *I^2^* = 97%) (Figure 3B). The positive association between dentition defects and the risk of Alzheimer's disease remained consistent across most subgroups.

### Publication bias and sensitivity analysis

Visual inspection of the funnel plot revealed some asymmetry (Supplementary Figure S2), suggesting potential publication bias or small-study effects. However, Egger's test (*P* = 0.12) and Begg's test (*P* = 0.15) did not reach statistical significance. This apparent discordance between visual and statistical assessments is not uncommon in meta-analyses with a limited number of studies (*k* = 14), as statistical tests for funnel plot asymmetry have low power when fewer than 10–20 studies are included (26). To further assess the robustness of our findings, we conducted a trim-and-fill analysis, which imputed two hypothetical missing studies to achieve funnel plot symmetry. The trim-and-fill analysis imputed two hypothetical missing studies to achieve funnel plot symmetry. Under the random-effects model, the adjusted pooled estimate (on the log scale) was 0.331 (95% CI: 0.102–0.561), corresponding to an odds ratio of 1.39 (95% CI: 1.11–1.75) after exponentiation (Supplementary Figure S4), which is virtually identical to the original estimate.

Further ensuring the robustness and dependability of our primary outcomes, sensitivity analyses were conducted by sequentially excluding individual studies. Sensitivity analysis showed that our results were stable and reliable, with pooled ORs ranging from 1.09 (95% CI: 1.03–1.15) to 1.14 (95% CI: 1.05–1.24) for the relationship between AD and DD (Supplementary Figure S3), indicating that no single study disproportionately influenced the overall estimate.

## Quality assessment

The GRADE tool was used to evaluate the quality of evidence of cohort studies. All the included studies were observational cohort studies, so the initial level of evidence was moderate (27). As there was greater heterogeneity in the risk of AD (*I^2^* = 97%), downgrades were given. Ultimately, the risk of AD was evaluated as low by the GRADE tool (Supplementary Table S5).

## Discussion

This systematic review and meta-analysis provided several novel contributions to the growing literature on oral health and neurodegenerative disease. First, while previous meta-analyses have focused broadly on tooth loss and cognitive impairment or dementia, our study specifically examines Alzheimer's disease as a distinct outcome, addressing potential etiological differences between AD and other dementia subtypes. A recent meta-analysis reported that tooth loss increases dementia risk approximately 3.64-fold, but did not specifically examine AD as a separate outcome (28). By focusing specifically on AD, we provide more targeted evidence for the relationship between oral health and this specific neurodegenerative condition.

By including studies published up to 2026, we provide the most current synthesis of evidence, capturing recent large-scale cohort studies not available in earlier reviews. Notably, our inclusion of studies examining occlusal function and prosthetic rehabilitation aligns with emerging evidence from Mendelian randomization studies demonstrating causal relationships between occlusion dysfunction and AD 8.

Our subgroup analysis identified notable geographic variation in the DD-AD association, with stronger effect sizes observed in Asian populations compared to European and North American cohorts. This finding has not been systematically documented previously and suggests potential gene-environment interactions or cultural differences in oral health practices and dietary patterns. The APOE4 (Apolipoprotein E4) genotype, the primary genetic marker for late-onset AD, has been shown to interact with dental occlusion deficiency as an independent risk factor for AD29, suggesting that genetic predisposition may modulate the oral health-AD relationship across populations with different APOE allele frequencies.

We systematically integrated mechanistic pathways—chronic inflammation, nutritional deficiency, microbiome dysbiosis, and cognitive reserve—into a cohesive framework linking peripheral oral pathology to central neurodegeneration. This framework draws on recent evidence demonstrating that inflammatory diets modulate both oral and gut microbiomes in AD patients, with associated changes in systemic inflammatory markers (7). This review positions DD within the broader context of modifiable risk factors for dementia, providing an evidence base for including oral health in multi-domain prevention interventions. Recent evidence summaries have identified key components of oral health management for cognitively impaired older adults, and our findings support the integration of these components into comprehensive dementia prevention strategies.

Oral health, particularly the presence of dental defects, can not only result in poor nutritional intake but is also associated with cognitive decline, a condition particularly pronounced in patients with AD. Singhrao et al. highlighted a correlation between oral inflammation, tooth loss, and other risk factors with the progression of AD (29). Furthermore, a case-control study by Holmer et al. suggested a potential link between periodontal inflammation and an increased risk of AD (30). Consistent with previous research, our study reveals a significant association between AD and dental defects, with individuals experiencing dental defects being at a higher risk of developing AD (OR: 1.38; 95% CI: 1.09–1.74). To ensure the rigor of our findings, we conducted thorough analyses to address potential publication bias, affirming the reliability of our results.

The meta-analysis revealed substantial heterogeneity across studies (*I^2^* = 97%, Tau^2^ = 0.15, *P* for heterogeneity < 0.001), indicating considerable variability in effect estimates beyond what would be expected by chance alone. This degree of heterogeneity was anticipated given the diversity in study designs, populations, exposure definitions, diagnostic criteria for dementia, and adjustments for confounding variables across the included studies. Such variability suggests that the true association between dental diseases (DD) and Alzheimer's disease (AD) risk may differ across populations and study contexts rather than representing a single uniform effect (10). To explore potential sources of heterogeneity, we conducted pre-specified subgroup analyses by study design and geographic region. However, substantial residual heterogeneity remained after subgroup analyses, indicating that additional factors—such as differences in exposure assessment methods, diagnostic criteria for AD, and variations in confounder adjustment—may contribute to the observed variability. To better reflect the uncertainty associated with this heterogeneity, we calculated prediction intervals to estimate the range within which the effect size of a future comparable study may lie. Unlike conventional confidence intervals, prediction intervals account for between-study heterogeneity and therefore provide a more realistic representation of the dispersion of effect estimates. The wide prediction interval suggests that the magnitude of the association may vary substantially across different settings and populations. Accordingly, although the pooled odds ratio indicates a positive association between dental diseases and dementia risk, this summary estimate should be interpreted cautiously and viewed as an average effect rather than a precise estimate applicable to all populations.

Publication bias was explored using funnel plot inspection and the trim-and-fill method. However, the interpretation of these analyses should be made cautiously given the substantial between-study heterogeneity observed in this meta-analysis (*I^2^* = 97%). The trim-and-fill procedure assumes that studies estimate a single underlying true effect size, an assumption that may not hold in the presence of extreme heterogeneity across studies. In such contexts, funnel plot asymmetry may arise not only from publication bias but also from alternative sources, including small-study effects, differences in study design, population characteristics, outcome definitions, or residual confounding. Smaller studies may report larger effect estimates due to methodological variability, selective reporting, or differences in adjustment for confounders. Therefore, while the trim-and-fill analysis provides an exploratory assessment of potential publication bias, its results should be interpreted cautiously. The presence of substantial heterogeneity limits the reliability of statistical methods designed to detect or correct for publication bias, and the observed asymmetry may reflect genuine between-study variability rather than missing unpublished studies.

### Potential mechanism

The potential biological mechanisms linking dental diseases (DD) and Alzheimer's disease (AD) are complex and heterogeneous, as dental diseases include both inflammatory conditions such as periodontitis and structural outcomes such as tooth loss. Importantly, many mechanistic hypotheses have been derived primarily from periodontal disease research rather than from studies directly examining tooth loss.

Periodontitis has been proposed to influence neurodegeneration through systemic inflammatory pathways. Chronic periodontal infection can induce low-grade systemic inflammation characterized by elevated inflammatory mediators such as IL-1β, IL-6, TNF-α, and C-reactive protein. These inflammatory responses may compromise blood–brain barrier integrity, activate microglia, and promote neuroinflammatory processes associated with amyloid-β accumulation and tau hyperphosphorylation (31). In addition, periodontal pathogens including Porphyromonas gingivalis and Treponema denticola, as well as their virulence factors such as lipopolysaccharide and gingipains, have been detected in experimental and neuropathological studies and are hypothesized to contribute to neuroinflammation (31). However, these inflammatory and microbiological mechanisms are primarily supported by the periodontal disease literature. Tooth loss, which may result from multiple causes including dental caries, trauma, or periodontal disease, does not necessarily involve chronic inflammation. Therefore, the direct applicability of these mechanisms to tooth loss remains uncertain, and mechanistic evidence specifically linking tooth loss to neurodegenerative processes is currently limited.

Alternative pathways proposed for tooth loss involve functional and behavioral changes. Loss of teeth may impair masticatory efficiency, leading to dietary modifications and reduced intake of neuroprotective nutrients such as omega-3 fatty acids, B vitamins, vitamin D, and antioxidants. These nutritional changes may influence the gut microbiome and systemic inflammatory status through the gut–brain axis (28). In addition, masticatory dysfunction and oral impairment may affect cognitive reserve through reduced social interaction, decreased participation in cognitively stimulating activities, and altered cerebral perfusion. Observational studies have also reported associations between tooth loss and hippocampal atrophy, although causal mechanisms remain to be clarified (28). Finally, genetic susceptibility may interact with oral health conditions. For example, the APOE ε4 allele, a major genetic risk factor for late-onset AD, may modify the relationship between oral health status and cognitive decline, although evidence in this area remains preliminary (32).

### Clinical implications

Oral health assessment may warrant consideration within the broader context of dementia risk research. The observed association between oral health conditions and dementia suggests that oral health could represent a potentially modifiable factor related to cognitive aging; however, current evidence remains limited and primarily observational. Therefore, these findings should be interpreted as indicative of association rather than causation. Given the high global prevalence of dental diseases among older adults—particularly severe periodontitis and tooth loss—further investigation is warranted to clarify whether these conditions contribute to dementia risk or reflect shared underlying mechanisms. Longitudinal cohort studies and well-designed interventional research are needed to better determine the directionality and potential biological pathways linking oral health and cognitive outcomes. Although prosthetic rehabilitation, including dentures and dental implants, has been hypothesized to influence mechanisms such as masticatory function, cerebral perfusion, and systemic inflammation, current evidence does not support definitive conclusions regarding their impact on dementia prevention. Consequently, these interventions should not yet be considered established strategies for cognitive protection. Importantly, oral diseases disproportionately affect socioeconomically disadvantaged populations and groups with limited access to dental care. This pattern suggests that oral health may reflect broader social and health inequalities associated with cognitive aging. Future public health and epidemiological research may benefit from incorporating oral health indicators into studies of cognitive decline to better understand these complex relationships.

### Study limitations

This study has several limitations. Firstly, the mechanistic pathways discussed below are primarily derived from research on periodontitis, a common cause of tooth loss. However, it is important to recognize that tooth loss can also result from caries, trauma, or other conditions not necessarily accompanied by chronic inflammation. Therefore, while inflammation and microbial translocation are plausible links in the context of periodontitis-associated tooth loss, direct evidence for these mechanisms in tooth loss from other causes is lacking. Alternative mechanisms, such as impaired mastication leading to nutritional changes and reduced cognitive stimulation, may play a more prominent role in non-inflammatory tooth loss. Second, some studies used retrospective designs, which may have introduced recall bias, highlighting the need for prospective research. Additionally, differences in study populations, methodologies, and diagnostic criteria contributed to heterogeneity, potentially limiting the generalizability of the findings. Furthermore, as all included studies were observational, residual confounding cannot be entirely ruled out, and the overall quality of evidence is lower compared to randomized controlled trials. Fourth, the combination of ORs, RRs, and HRs from different study designs may introduce bias. Although we assumed the rare disease assumption and conducted subgroup analyses showing consistent effects across designs, residual differences in confounding adjustment and design-specific biases cannot be entirely excluded. Future meta-analyses with larger numbers of studies could employ meta-regression to formally test for differences by study design or effect measure type. Fifth, the predominance of case-control studies (10 of 14, 71%) in our meta-analysis warrants careful consideration of design-specific biases. Case-control designs are inherently susceptible to: Selection bias: Cases (AD patients) and controls may be selected from different source populations, leading to differential exposure distributions unrelated to true causal effects. For example, controls recruited from hospital settings may have different oral health profiles than community-dwelling controls, potentially biasing the observed association (33). Exposure assessment (DD history) often relies on self-report or proxy-report in case-control studies. AD patients may have difficulty recalling past dental history due to cognitive impairment, while caregivers may provide incomplete information. This differential recall between cases and controls could bias estimates in either direction. Furthermore, in case-control studies, the temporal relationship between exposure and outcome cannot be established with certainty. Poor oral health in AD patients may reflect pre-diagnostic cognitive decline affecting self-care (reverse causality) rather than contributing to disease onset (13). Prevalent case designs may include only surviving AD patients, potentially underestimating the association if DD is associated with mortality. To partially address these concerns, we conducted subgroup analysis by study design (Figure 3A), which showed consistent effect estimates between case-control (OR  = 1.35) and cohort studies (OR = 1.42). However, the small number of cohort studies (*n* = 4) limits the power of this comparison. Future prospective cohort studies with incident AD cases, standardized exposure assessment, and comprehensive confounder adjustment are urgently needed to overcome the inherent limitations of case-control designs. Sixth, confounding by indication and shared risk factors cannot be excluded. Individuals with poor oral health may also have lower socioeconomic status, reduced access to healthcare, higher rates of smoking, poorer nutrition, and greater burden of chronic diseases—all factors independently associated with AD risk. While included studies adjusted for various confounders, residual confounding remains possible. Future studies with rigorous control for socioeconomic factors, health behaviors, and comorbidities are needed to better isolate the independent association between DD and AD.

## Conclusion

This systematic review and meta-analysis suggest a significant association between dentition defects and increased risk of Alzheimer's disease. However, due to the low quality of the available evidence and substantial heterogeneity, these findings should be interpreted with caution. Oral health may represent a modifiable factor warranting further investigation in well-designed prospective studies and randomized controlled trials to establish causality and inform potential preventive strategies.

## Data Availability

The original contributions presented in the study are included in the article/Supplementary Material, further inquiries can be directed to the corresponding author.
