# Supplementary material for: Association between dentition defects and Alzheimer’s disease risk: a systematic review and meta-analysis
Source: Front Dent Med. 2026 Apr 20;7:1783171. doi: 10.3389/fdmed.2026.1783171 (PMC13136256; doi:10.3389/fdmed.2026.1783171)
Supplement: Supplementary file 1 [file Table2.docx]

**Supplementary Table S1: Article content checklist**

| **Section/topic** | | **#** | **Checklist item** | **Reported on page #** |
| --- | --- | --- | --- | --- |
| **TITLE** | | | |  |
| Title | | 1 | Identify the report as a systematic review, meta-analysis, or both. | 1 |
| **ABSTRACT** | | | |  |
| Structured summary | | 2 | Provide a structured summary including, as applicable: background; objectives; data sources; study eligibility criteria, participants, and interventions; study appraisal and synthesis methods; results; limitations; conclusions and implications of key findings; systematic review registration number. | 1 |
| **INTRODUCTION** | | | |  |
| Rationale | | 3 | Describe the rationale for the review in the context of what is already known. | 2 |
| Objectives | | 4 | Provide an explicit statement of questions being addressed with reference to participants, interventions, comparisons, outcomes, and study design (PICOS). | 2 |
| **METHODS** | | | |  |
| Protocol and registration | | 5 | Indicate if a review protocol exists, if and where it can be accessed (e.g., Web address), and, if available, provide registration information including registration number. | 3 |
| Eligibility criteria | | 6 | Specify study characteristics (e.g., PICOS, length of follow-up) and report characteristics (e.g., years considered, language, publication status) used as criteria for eligibility, giving rationale. | 4 |
| Information sources | | 7 | Describe all information sources (e.g., databases with dates of coverage, contact with study authors to identify additional studies) in the search and date last searched. | 4 |
| Search | | 8 | Present full electronic search strategy for at least one database, including any limits used, such that it could be repeated. | 4 |
| Study selection | | 9 | State the process for selecting studies (i.e., screening, eligibility, included in systematic review, and, if applicable, included in the meta-analysis). | 4 |
| Data collection process | | 10 | Describe method of data extraction from reports (e.g., piloted forms, independently, in duplicate) and any processes for obtaining and confirming data from investigators. | 5 |
| Data items | | 11 | List and define all variables for which data were sought (e.g., PICOS, funding sources) and any assumptions and simplifications made. | 5 |
| Risk of bias in individual studies | | 12 | Describe methods used for assessing risk of bias of individual studies (including specification of whether this was done at the study or outcome level), and how this information is to be used in any data synthesis. | 6 |
| Summary measures | | 13 | State the principal summary measures (e.g., risk ratio, difference in means). | 6 |
| Synthesis of results | | 14 | Describe the methods of handling data and combining results of studies, if done, including measures of consistency (e.g., I^2^) for each meta-analysis. | 7 |
| Risk of bias across studies | | 15 | Specify any assessment of risk of bias that may affect the cumulative evidence (e.g., publication bias, selective reporting within studies). | 7 |
| Additional analyses | | 16 | Describe methods of additional analyses (e.g., sensitivity or subgroup analyses, meta-regression), if done, indicating which were pre-specified. | 7 |
| **RESULTS** | | | |  |
| Study selection | 17 | | Give numbers of studies screened, assessed for eligibility, and included in the review, with reasons for exclusions at each stage, ideally with a flow diagram. | 7 |
| Study characteristics | 18 | | For each study, present characteristics for which data were extracted (e.g., study size, PICOS, follow-up period) and provide the citations. | 8 |
| Risk of bias within studies | 19 | | Present data on risk of bias of each study and, if available, any outcome level assessment (see item 12). | 9 |
| Results of individual studies | 20 | | For all outcomes considered (benefits or harms), present, for each study: (a) simple summary data for each intervention group (b) effect estimates and confidence intervals, ideally with a forest plot. | 9 |
| Synthesis of results | 21 | | Present results of each meta-analysis done, including confidence intervals and measures of consistency. | 10 |
| Risk of bias across studies | 22 | | Present results of any assessment of risk of bias across studies (see Item 15). | 10 |

**Supplementary Table S2**: **Detailed description of the search strategy**

| Datebase | Search | Query |
| --- | --- | --- |
| cochrance | #1 | (Edentulous Mouth):ab,ti,kw OR (Edentulous Mouths):ab,ti,kw OR (Mouth, Toothless):ab,ti,kw OR (Toothless Mouth):ab,ti,kw OR (Tooth loss):ab,ti,kw OR (Dentition defect):ab,ti,kw OR (Edentulous):ab,ti,kw OR (Dental loss):ab,ti,kw OR (Periodontal disease):ab,ti,kw OR (gingiva bleeding):ab,ti,kw OR (gingiva overgrowth):ab,ti,kw OR (gingiva fibromatosis):ab,ti,kw OR (gingiva hyperplasia):ab,ti,kw OR (gingiva hypertrophy):ab,ti,kw OR (gingiva pain):ab,ti,kw OR (gingiva tumor):ab,ti,kw OR (gingiva ulcer):ab,ti,kw OR (gingival cyst):ab,ti,kw OR (gingivitis):ab,ti,kw OR (Vincent stomatitis):ab,ti,kw OR (peri-implant mucositis):ab,ti,kw OR (periimplantitis):ab,ti,kw OR (periodontal cyst):ab,ti,kw OR (periodontosis):ab,ti,kw OR (periodontitis):ab,ti,kw OR (aggressive periodontitis):ab,ti,kw OR (chronic periodontitis):ab,ti,kw OR (gingivitis):ab,ti,kw OR (Papillon Lefevre syndrome):ab,ti,kw OR (periodontal abscess):ab,ti,kw OR (periodontal pocket):ab,ti,kw OR (Vincent stomatitis):ab,ti,kw OR (periapical abscess):ab,ti,kw OR (tooth periapical disease):ab,ti,kw OR (periapical abscess):ab,ti,kw |
|  | #2 | (Alzheimer Dementia):ab,ti,kw OR (Alzheimer Dementias):ab,ti,kw OR (Dementia, Alzheimer):ab,ti,kw OR (Alzheimer's Disease):ab,ti,kw OR (Dementia, Senile):ab,ti,kw OR (Senile Dementia):ab,ti,kw OR (Dementia, Alzheimer Type):ab,ti,kw OR (Alzheimer Type Dementia):ab,ti,kw OR (Alzheimer-Type Dementia (ATD)):ab,ti,kw OR (Alzheimer Type Dementia (ATD)):ab,ti,kw OR (Dementia, Alzheimer-Type (ATD)):ab,ti,kw OR (Alzheimer Type Senile Dementia):ab,ti,kw OR (Primary Senile Degenerative Dementia):ab,ti,kw OR (Dementia, Primary Senile Degenerative):ab,ti,kw OR (Alzheimer Sclerosis):ab,ti,kw OR (Sclerosis, Alzheimer):ab,ti,kw OR (Alzheimer Syndrome):ab,ti,kw OR (Alzheimer's Diseases):ab,ti,kw OR (Alzheimer Diseases):ab,ti,kw OR (Alzheimers Diseases):ab,ti,kw OR (Senile Dementia, Alzheimer Type):ab,ti,kw OR (Acute Confusional Senile Dementia):ab,ti,kw OR (Senile Dementia, Acute Confusional):ab,ti,kw OR (Dementia, Presenile):ab,ti,kw OR (Presenile Dementia):ab,ti,kw OR (Alzheimer Disease, Late Onset):ab,ti,kw OR (Late Onset Alzheimer Disease):ab,ti,kw OR (Alzheimer's Disease, Focal Onset):ab,ti,kw OR (Focal Onset Alzheimer's Disease):ab,ti,kw OR (Familial Alzheimer Disease (FAD)):ab,ti,kw OR (Alzheimer Disease, Familial (FAD)):ab,ti,kw OR (Familial Alzheimer Diseases (FAD)):ab,ti,kw OR (Alzheimer Disease, Early Onset):ab,ti,kw OR (Early Onset Alzheimer Disease):ab,ti,kw OR (Presenile Alzheimer Dementia):ab,ti,kw |
|  | #3 | #1 AND #2 |
| Embase | #1 | tooth AND periapical AND disease OR (periapical AND abscess) OR (periodontal AND pocket) OR (periodontal AND abscess) OR 'papillon lefevre syndrome' OR (chronic AND periodontitis) OR (aggressive AND periodontitis) OR periodontitis OR periodontosis OR (periodontal AND cyst) OR periimplantitis OR ('peri implant' AND mucositis) OR (vincent AND stomatitis) OR gingivitis OR (gingival AND cyst) OR (gingiva AND ulcer) OR (gingiva AND tumor) OR (gingiva AND pain) OR (gingiva AND hypertrophy) OR (gingiva AND hyperplasia) OR (gingiva AND fibromatosis) OR (gingiva AND overgrowth) OR (gingiva AND bleeding) OR (periodontal AND disease) OR (dental AND loss) OR edentulous OR (dentition AND defect) OR (tooth AND loss) OR (toothless AND mouth) OR (mouth, AND toothless) OR (edentulous AND mouths) OR (edentulous AND mouth) |
|  | #2 | 'presenile alzheimer dementia' OR (presenile AND alzheimer AND ('dementia'/exp OR dementia)) OR 'early onset alzheimer disease'/exp OR 'early onset alzheimer disease' OR (early AND onset AND alzheimer AND ('disease'/exp OR disease)) OR 'alzheimer disease, early onset' OR (alzheimer AND ('disease,'/exp OR disease,) AND early AND onset) OR 'familial alzheimer diseases' OR (familial AND alzheimer AND ('diseases'/exp OR diseases) AND ('fad'/exp OR fad)) OR 'alzheimer disease, familial' OR (alzheimer AND ('disease,'/exp OR disease,) AND familial AND ('fad'/exp OR fad)) OR 'familial alzheimer disease'/exp OR 'familial alzheimer disease' OR (familial AND alzheimer AND ('disease'/exp OR disease) AND ('fad'/exp OR fad)) OR 'focal onset alzheimers disease' OR (focal AND onset AND alzheimers AND ('disease'/exp OR disease)) OR 'alzheimers disease, focal onset' OR (alzheimers AND ('disease,'/exp OR disease,) AND focal AND onset) OR 'late onset alzheimer disease'/exp OR 'late onset alzheimer disease' OR (late AND onset AND alzheimer AND ('disease'/exp OR disease)) OR 'alzheimer disease, late onset' OR (alzheimer AND ('disease,'/exp OR disease,) AND late AND onset) OR 'presenile dementia'/exp OR 'presenile dementia' OR (presenile AND ('dementia'/exp OR dementia)) OR 'dementia, presenile'/exp OR 'dementia, presenile' OR (('dementia,'/exp OR dementia,) AND presenile) OR 'senile dementia, acute confusional' OR (senile AND ('dementia,'/exp OR dementia,) AND acute AND confusional) OR 'acute confusional senile dementia' OR (acute AND confusional AND senile AND ('dementia'/exp OR dementia)) OR 'senile dementia, alzheimer type' OR (senile AND ('dementia,'/exp OR dementia,) AND alzheimer AND type) OR 'alzheimer diseases' OR (alzheimer AND ('diseases'/exp OR diseases)) OR 'alzheimers diseases' OR (alzheimers AND ('diseases'/exp OR diseases)) OR 'alzheimer syndrome'/exp OR 'alzheimer syndrome' OR (alzheimer AND ('syndrome'/exp OR syndrome)) OR 'sclerosis, alzheimer' OR (('sclerosis,'/exp OR sclerosis,) AND alzheimer) OR 'alzheimer sclerosis'/exp OR 'alzheimer sclerosis' OR (alzheimer AND ('sclerosis'/exp OR sclerosis)) OR 'dementia, primary senile degenerative' OR (('dementia,'/exp OR dementia,) AND primary AND senile AND degenerative) OR 'primary senile degenerative dementia' OR (primary AND senile AND degenerative AND ('dementia'/exp OR dementia)) OR 'alzheimer type senile dementia' OR (alzheimer AND type AND senile AND ('dementia'/exp OR dementia)) OR 'dementia, alzheimer-type' OR (('dementia,'/exp OR dementia,) AND 'alzheimer type' AND atd) OR (alzheimer AND type AND ('dementia'/exp OR dementia) AND atd) OR 'alzheimer-type dementia' OR ('alzheimer type' AND ('dementia'/exp OR dementia) AND atd) OR 'alzheimer type dementia' OR (alzheimer AND type AND ('dementia'/exp OR dementia)) OR 'dementia, alzheimer type' OR (('dementia,'/exp OR dementia,) AND alzheimer AND type) OR 'senile dementia'/exp OR 'senile dementia' OR (senile AND ('dementia'/exp OR dementia)) OR 'dementia, senile'/exp OR 'dementia, senile' OR (('dementia,'/exp OR dementia,) AND senile) OR 'alzheimers disease'/exp OR 'alzheimers disease' OR (alzheimers AND ('disease'/exp OR disease)) OR 'dementia, alzheimer'/exp OR 'dementia, alzheimer' OR (('dementia,'/exp OR dementia,) AND alzheimer) OR 'alzheimer dementias' OR (alzheimer AND dementias) OR 'alzheimer dementia'/exp OR 'alzheimer dementia' OR (alzheimer AND ('dementia'/exp OR dementia)) |
|  | #3 | (tooth AND periapical AND disease OR (periapical AND abscess) OR (periodontal AND pocket) OR (periodontal AND abscess) OR 'papillon lefevre syndrome' OR (chronic AND periodontitis) OR (aggressive AND periodontitis) OR periodontitis OR periodontosis OR (periodontal AND cyst) OR periimplantitis OR ('peri implant' AND mucositis) OR (vincent AND stomatitis) OR gingivitis OR (gingival AND cyst) OR (gingiva AND ulcer) OR (gingiva AND tumor) OR (gingiva AND pain) OR (gingiva AND hypertrophy) OR (gingiva AND hyperplasia) OR (gingiva AND fibromatosis) OR (gingiva AND overgrowth) OR (gingiva AND bleeding) OR (periodontal AND disease) OR (dental AND loss) OR edentulous OR (dentition AND defect) OR (tooth AND loss) OR (toothless AND mouth) OR (mouth, AND toothless) OR (edentulous AND mouths) OR (edentulous AND mouth)) AND ('presenile alzheimer dementia' OR (presenile AND alzheimer AND ('dementia'/exp OR dementia)) OR 'early onset alzheimer disease'/exp OR 'early onset alzheimer disease' OR (early AND onset AND alzheimer AND ('disease'/exp OR disease)) OR 'alzheimer disease, early onset' OR (alzheimer AND ('disease,'/exp OR disease,) AND early AND onset) OR 'familial alzheimer diseases' OR (familial AND alzheimer AND ('diseases'/exp OR diseases) AND ('fad'/exp OR fad)) OR 'alzheimer disease, familial' OR (alzheimer AND ('disease,'/exp OR disease,) AND familial AND ('fad'/exp OR fad)) OR 'familial alzheimer disease'/exp OR 'familial alzheimer disease' OR (familial AND alzheimer AND ('disease'/exp OR disease) AND ('fad'/exp OR fad)) OR 'focal onset alzheimers disease' OR (focal AND onset AND alzheimers AND ('disease'/exp OR disease)) OR 'alzheimers disease, focal onset' OR (alzheimers AND ('disease,'/exp OR disease,) AND focal AND onset) OR 'late onset alzheimer disease'/exp OR 'late onset alzheimer disease' OR (late AND onset AND alzheimer AND ('disease'/exp OR disease)) OR 'alzheimer disease, late onset' OR (alzheimer AND ('disease,'/exp OR disease,) AND late AND onset) OR 'presenile dementia'/exp OR 'presenile dementia' OR (presenile AND ('dementia'/exp OR dementia)) OR 'dementia, presenile'/exp OR 'dementia, presenile' OR (('dementia,'/exp OR dementia,) AND presenile) OR 'senile dementia, acute confusional' OR (senile AND ('dementia,'/exp OR dementia,) AND acute AND confusional) OR 'acute confusional senile dementia' OR (acute AND confusional AND senile AND ('dementia'/exp OR dementia)) OR 'senile dementia, alzheimer type' OR (senile AND ('dementia,'/exp OR dementia,) AND alzheimer AND type) OR 'alzheimer diseases' OR (alzheimer AND ('diseases'/exp OR diseases)) OR 'alzheimers diseases' OR (alzheimers AND ('diseases'/exp OR diseases)) OR 'alzheimer syndrome'/exp OR 'alzheimer syndrome' OR (alzheimer AND ('syndrome'/exp OR syndrome)) OR 'sclerosis, alzheimer' OR (('sclerosis,'/exp OR sclerosis,) AND alzheimer) OR 'alzheimer sclerosis'/exp OR 'alzheimer sclerosis' OR (alzheimer AND ('sclerosis'/exp OR sclerosis)) OR 'dementia, primary senile degenerative' OR (('dementia,'/exp OR dementia,) AND primary AND senile AND degenerative) OR 'primary senile degenerative dementia' OR (primary AND senile AND degenerative AND ('dementia'/exp OR dementia)) OR 'alzheimer type senile dementia' OR (alzheimer AND type AND senile AND ('dementia'/exp OR dementia)) OR 'dementia, alzheimer-type' OR (('dementia,'/exp OR dementia,) AND 'alzheimer type' AND atd) OR (alzheimer AND type AND ('dementia'/exp OR dementia) AND atd) OR 'alzheimer-type dementia' OR ('alzheimer type' AND ('dementia'/exp OR dementia) AND atd) OR 'alzheimer type dementia' OR (alzheimer AND type AND ('dementia'/exp OR dementia)) OR 'dementia, alzheimer type' OR (('dementia,'/exp OR dementia,) AND alzheimer AND type) OR 'senile dementia'/exp OR 'senile dementia' OR (senile AND ('dementia'/exp OR dementia)) OR 'dementia, senile'/exp OR 'dementia, senile' OR (('dementia,'/exp OR dementia,) AND senile) OR 'alzheimers disease'/exp OR 'alzheimers disease' OR (alzheimers AND ('disease'/exp OR disease)) OR 'dementia, alzheimer'/exp OR 'dementia, alzheimer' OR (('dementia,'/exp OR dementia,) AND alzheimer) OR 'alzheimer dementias' OR (alzheimer AND dementias) OR 'alzheimer dementia'/exp OR 'alzheimer dementia' OR (alzheimer AND ('dementia'/exp OR dementia))) |
| Web of science | #1 | ((((((((((((((((((((((((((((((((((TS=(periapical abscess)) OR TS=(tooth periapical disease)) OR TS=(periapical abscess)) OR TS=(Vincent stomatitis)) OR TS=(periodontal pocket)) OR TS=(periodontal abscess)) OR TS=(Papillon Lefevre syndrome)) OR TS=(gingivitis)) OR TS=(chronic periodontitis)) OR TS=(aggressive periodontitis)) OR TS=(periodontitis)) OR TS=(periodontosis)) OR TS=(periodontal cyst)) OR TS=(periimplantitis)) OR TS=(peri-implant mucositis)) OR TS=(Vincent stomatitis)) OR TS=(gingivitis)) OR TS=(gingival cyst)) OR TS=(gingiva ulcer)) OR TS=(gingiva tumor)) OR TS=(gingiva pain)) OR TS=(gingiva hypertrophy)) OR TS=(gingiva hyperplasia)) OR TS=(gingiva fibromatosis)) OR TS=(gingiva overgrowth)) OR TS=(gingiva bleeding)) OR TS=(Periodontal disease)) OR TS=(Dental loss)) OR TS=(Edentulous)) OR TS=(Dentition defect)) OR TS=(Tooth loss)) OR TS=(Toothless Mouth)) OR TS=(Mouth, Toothless)) OR TS=(Edentulous Mouths)) OR TS=(Edentulous Mouth) and Preprint Citation Index (Exclude – Database) |
|  | #2 | ((((((((((((((((((((((((((((((((((TS=(Presenile Alzheimer Dementia)) OR TS=(Early Onset Alzheimer Disease)) OR TS=(Alzheimer Disease, Early Onset)) OR TS=(Familial Alzheimer Diseases (FAD))) OR TS=(Alzheimer Disease, Familial (FAD))) OR TS=(Familial Alzheimer Disease (FAD))) OR TS=(Focal Onset Alzheimer's Disease)) OR TS=(Alzheimer's Disease, Focal Onset)) OR TS=(Late Onset Alzheimer Disease)) OR TS=(Alzheimer Disease, Late Onset)) OR TS=(Presenile Dementia)) OR TS=(Dementia, Presenile)) OR TS=(Senile Dementia, Acute Confusional)) OR TS=(Acute Confusional Senile Dementia)) OR TS=(Senile Dementia, Alzheimer Type)) OR TS=(Alzheimers Diseases)) OR TS=(Alzheimer Diseases)) OR TS=(Alzheimer's Diseases)) OR TS=(Alzheimer Syndrome)) OR TS=(Sclerosis, Alzheimer)) OR TS=(Alzheimer Sclerosis)) OR TS=(Dementia, Primary Senile Degenerative)) OR TS=(Primary Senile Degenerative Dementia)) OR TS=(Alzheimer Type Senile Dementia)) OR TS=(Dementia, Alzheimer-Type (ATD))) OR TS=(Alzheimer Type Dementia (ATD))) OR TS=(Alzheimer-Type Dementia (ATD))) OR TS=(Alzheimer Type Dementia)) OR TS=(Dementia, Alzheimer Type)) OR TS=(Senile Dementia)) OR TS=(Dementia, Senile)) OR TS=(Alzheimer's Disease)) OR TS=(Dementia, Alzheimer)) OR TS=(Alzheimer Dementias)) OR TS=(Alzheimer Dementia) and Preprint Citation Index (Exclude – Database) |
|  | #3 | #2 AND #1 and Preprint Citation Index (Exclude – Database) |
| PubMed | #1 | ((((((((((((((((((((((((((((((((((Periodontal disease) OR (gingiva bleeding)) OR (gingiva overgrowth)) OR (gingiva fibromatosis)) OR (gingiva hyperplasia)) OR (gingiva hypertrophy)) OR (gingiva pain)) OR (gingiva tumor)) OR (gingiva ulcer)) OR (gingival cyst)) OR (gingivitis)) OR (Vincent stomatitis)) OR (peri-implant mucositis)) OR (periimplantitis)) OR (periodontal cyst)) OR (periodontosis)) OR (periodontitis)) OR (aggressive periodontitis)) OR (chronic periodontitis)) OR (gingivitis)) OR (Papillon Lefevre syndrome)) OR (periodontal abscess)) OR (periodontal pocket)) OR (Vincent stomatitis)) OR (periapical abscess)) OR (tooth periapical disease)) OR (periapical abscess)) OR (Edentulous Mouth)) OR (Edentulous Mouths)) OR (Mouth, Toothless)) OR (Toothless Mouth)) OR (Tooth loss)) OR (Dentition defect)) OR (Edentulous)) OR (Dental loss) |
|  | #2 | ((((((((((((((((((((((((((((((((((Alzheimer Dementia) OR (Alzheimer Dementias)) OR (Dementia, Alzheimer)) OR (Alzheimer's Disease)) OR (Dementia, Senile)) OR (Senile Dementia)) OR (Dementia, Alzheimer Type)) OR (Alzheimer Type Dementia)) OR (Alzheimer-Type Dementia (ATD))) OR (Alzheimer Type Dementia (ATD))) OR (Dementia, Alzheimer-Type (ATD))) OR (Alzheimer Type Senile Dementia)) OR (Primary Senile Degenerative Dementia)) OR (Dementia, Primary Senile Degenerative)) OR (Alzheimer Sclerosis)) OR (Sclerosis, Alzheimer)) OR (Alzheimer Syndrome)) OR (Alzheimer's Diseases)) OR (Alzheimer Diseases)) OR (Alzheimers Diseases)) OR (Senile Dementia, Alzheimer Type)) OR (Acute Confusional Senile Dementia)) OR (Senile Dementia, Acute Confusional)) OR (Dementia, Presenile)) OR (Presenile Dementia)) OR (Alzheimer Disease, Late Onset)) OR (Late Onset Alzheimer Disease)) OR (Alzheimer's Disease, Focal Onset)) OR (Focal Onset Alzheimer's Disease)) OR (Familial Alzheimer Disease (FAD))) OR (Alzheimer Disease, Familial (FAD))) OR (Familial Alzheimer Diseases (FAD))) OR (Alzheimer Disease, Early Onset)) OR (Early Onset Alzheimer Disease)) OR (Presenile Alzheimer Dementia) |
|  | #3 | (((((((((((((((((((((((((((((((((((Periodontal disease) OR (gingiva bleeding)) OR (gingiva overgrowth)) OR (gingiva fibromatosis)) OR (gingiva hyperplasia)) OR (gingiva hypertrophy)) OR (gingiva pain)) OR (gingiva tumor)) OR (gingiva ulcer)) OR (gingival cyst)) OR (gingivitis)) OR (Vincent stomatitis)) OR (peri-implant mucositis)) OR (periimplantitis)) OR (periodontal cyst)) OR (periodontosis)) OR (periodontitis)) OR (aggressive periodontitis)) OR (chronic periodontitis)) OR (gingivitis)) OR (Papillon Lefevre syndrome)) OR (periodontal abscess)) OR (periodontal pocket)) OR (Vincent stomatitis)) OR (periapical abscess)) OR (tooth periapical disease)) OR (periapical abscess)) OR (Edentulous Mouth)) OR (Edentulous Mouths)) OR (Mouth, Toothless)) OR (Toothless Mouth)) OR (Tooth loss)) OR (Dentition defect)) OR (Edentulous)) OR (Dental loss)) AND (((((((((((((((((((((((((((((((((((Alzheimer Dementia) OR (Alzheimer Dementias)) OR (Dementia, Alzheimer)) OR (Alzheimer's Disease)) OR (Dementia, Senile)) OR (Senile Dementia)) OR (Dementia, Alzheimer Type)) OR (Alzheimer Type Dementia)) OR (Alzheimer-Type Dementia (ATD))) OR (Alzheimer Type Dementia (ATD))) OR (Dementia, Alzheimer-Type (ATD))) OR (Alzheimer Type Senile Dementia)) OR (Primary Senile Degenerative Dementia)) OR (Dementia, Primary Senile Degenerative)) OR (Alzheimer Sclerosis)) OR (Sclerosis, Alzheimer)) OR (Alzheimer Syndrome)) OR (Alzheimer's Diseases)) OR (Alzheimer Diseases)) OR (Alzheimers Diseases)) OR (Senile Dementia, Alzheimer Type)) OR (Acute Confusional Senile Dementia)) OR (Senile Dementia, Acute Confusional)) OR (Dementia, Presenile)) OR (Presenile Dementia)) OR (Alzheimer Disease, Late Onset)) OR (Late Onset Alzheimer Disease)) OR (Alzheimer's Disease, Focal Onset)) OR (Focal Onset Alzheimer's Disease)) OR (Familial Alzheimer Disease (FAD))) OR (Alzheimer Disease, Familial (FAD))) OR (Familial Alzheimer Diseases (FAD))) OR (Alzheimer Disease, Early Onset)) OR (Early Onset Alzheimer Disease)) OR (Presenile Alzheimer Dementia)) |

**Supplementary Table S3: Studies excluded (n=25) with reasons**

| **Studies excluded** | **Reasons** |
| --- | --- |
| Cicciù, M 2013^1^ | Can't extract accurate data |
| De Souza Rolim 2014^2^ | Can't extract accurate data |
| Delwel 2018^3^ | Can't extract accurate data |
| Fereshtehnejad 2018^4^ | Can't extract accurate data |
| Fu 2022^5^ | Can't extract accurate data |
| Gil Montoya 2020^6^ | Can't extract accurate data |
| Han 2020^7^ | Can't extract accurate data |
| Hatipoglu 2011^8^ | Can't extract accurate data |
| Holmer 2021^9^ | Can't extract accurate data |
| Holmer 2022^10^ | Can't extract accurate data |
| Hugo 2007^11^ | Can't extract accurate data |
| Jones 2003^12^ | Can't extract accurate data |
| Kim 2007^13^ | Can't extract accurate data |
| Luo 2021^14^ | Can't extract accurate data |
| Malone 2022^15^ | Unable extract accurate data |
| Popovac 2022^16^ | Can't extract accurate data |
| Ship 1992^17^ | Unable extract accurate data |
| Ship 1994^18^ | Can't extract accurate data |
| Syrjälä 2012^19^ | Unable extract accurate data |
| Tan 2020^20^ | Can't extract accurate data |
| Tiisanoja 2019^21^ | Unable extract accurate data |
| Warren 1997^22^ | Unable extract accurate data |
| Yang 2021^23^ | Can't extract accurate data |
| Arrivé 2012^24^ | Unable extract accurate data |
| Choi 2019^25^ | Unable extract accurate data |

1. Cicciù, M.; Matacena, G.; Signorino, F.; Brugaletta, A.; Cicciù, A.; Bramanti, E., Relationship between oral health and its impact on the quality life of Alzheimer's disease patients: a supportive care trial. *International journal of clinical and experimental medicine* **2013,** *6* (9), 766-72.

2. de Souza Rolim, T.; Fabri, G. M.; Nitrini, R.; Anghinah, R.; Teixeira, M. J.; de Siqueira, J. T.; Cestari, J. A.; de Siqueira, S. R., Oral infections and orofacial pain in Alzheimer's disease: a case-control study. *J Alzheimers Dis* **2014,** *38* (4), 823-9.

3. Delwel, S.; Scherder, E. J. A.; Perez, R.; Hertogh, C.; Maier, A. B.; Lobbezoo, F., Oral function of older people with mild cognitive impairment or dementia. *Journal of oral rehabilitation* **2018,** *45* (12), 990-997.

4. Fereshtehnejad, S. M.; Garcia-Ptacek, S.; Religa, D.; Holmer, J.; Buhlin, K.; Eriksdotter, M.; Sandborgh-Englund, G., Dental care utilization in patients with different types of dementia: A longitudinal nationwide study of 58,037 individuals. *Alzheimer's & dementia : the journal of the Alzheimer's Association* **2018,** *14* (1), 10-19.

5. Fu, K. L.; Chiu, M. J.; Wara-Aswapati, N.; Yang, C. N.; Chang, L. C.; Guo, Y. L.; Ni, Y. H.; Chen, Y. W., Oral microbiome and serological analyses on association of Alzheimer's disease and periodontitis. *Oral diseases* **2022**.

6. Gil Montoya, J. A.; Barrios, R.; Sanchez-Lara, I.; Ramos, P.; Carnero, C.; Fornieles, F.; Montes, J.; Santana, S.; Luna, J. D.; Gonzalez-Moles, M. A., Systemic inflammatory impact of periodontitis on cognitive impairment. *Gerodontology* **2020,** *37* (1), 11-18.

7. Han, J. H.; Lee, H. J.; Han, J. W.; Suh, S. W.; Lee, J. R.; Byun, S.; Kim, K. S.; Kim, S. Y.; Lee, J. T.; Yoo, E.; Chang, N. H.; Kim, T. H.; Kim, K. W., Loss of Functional Dentition is Associated with Cognitive Impairment. *J Alzheimers Dis* **2020,** *73* (4), 1313-1320.

8. Hatipoglu, M. G.; Kabay, S. C.; Güven, G., The clinical evaluation of the oral status in Alzheimer-type dementia patients. *Gerodontology* **2011,** *28* (4), 302-6.

9. Holmer, J.; Aho, V.; Eriksdotter, M.; Paulin, L.; Pietiäinen, M.; Auvinen, P.; Schultzberg, M.; Pussinen, P. J.; Buhlin, K., Subgingival microbiota in a population with and without cognitive dysfunction. *Journal of oral microbiology* **2021,** *13* (1), 1854552.

10. Holmer, J.; Eriksdotter, M.; Häbel, H.; Hed Myrberg, I.; Jonsson, A.; Pussinen, P. J.; Garcia-Ptacek, S.; Jansson, L.; Sandborgh-Englund, G.; Buhlin, K., Periodontal conditions and incident dementia: A nationwide Swedish cohort study. *Journal of periodontology* **2022,** *93* (9), 1378-1386.

11. Hugo, F. N.; Hilgert, J. B.; Bertuzzi, D.; Padilha, D. M.; De Marchi, R. J., Oral health behaviour and socio-demographic profile of subjects with Alzheimer's disease as reported by their family caregivers. *Gerodontology* **2007,** *24* (1), 36-40.

12. Jones, J. A.; Lavallee, N.; Alman, J.; Sinclair, C.; Garcia, R. I., Caries incidence in patients with dementia. *Gerodontology* **1993,** *10* (2), 76-82.

13. Kim, J. M.; Stewart, R.; Prince, M.; Kim, S. W.; Yang, S. J.; Shin, I. S.; Yoon, J. S., Dental health, nutritional status and recent-onset dementia in a Korean community population. *International journal of geriatric psychiatry* **2007,** *22* (9), 850-5.

14. Luo, H.; Tan, C.; Adhikari, S.; Plassman, B. L.; Kamer, A. R.; Sloan, F. A.; Schwartz, M. D.; Qi, X.; Wu, B., Effects of the Co-occurrence of Diabetes Mellitus and Tooth Loss on Cognitive Function. *Current Alzheimer research* **2021,** *18* (13), 1023-1031.

15. Malone, J.; Jung, J.; Tran, L.; Zhao, C., odontal Disease and Risk of Dementia in Medicare Patients with Hepatitis C VirusPeri. *J Alzheimers Dis* **2022,** *85* (3), 1301-1308.

16. Popovac, A.; Mladenović, I.; Krunić, J.; Trifković, B.; Todorović, A.; Milašin, J.; Despotović, N.; Stančić, I., Apolipoprotein ɛ4 Allele and Dental Occlusion Deficiency as Risk Factors for Alzheimer's Disease. *J Alzheimers Dis* **2020,** *74* (3), 797-802.

17. Ship, J. A., Oral health of patients with Alzheimer's disease. *Journal of the American Dental Association (1939)* **1992,** *123* (1), 53-8.

18. Ship, J. A.; Puckett, S. A., Longitudinal study on oral health in subjects with Alzheimer's disease. *Journal of the American Geriatrics Society* **1994,** *42* (1), 57-63.

19. Syrjälä, A. M.; Ylöstalo, P.; Ruoppi, P.; Komulainen, K.; Hartikainen, S.; Sulkava, R.; Knuuttila, M., Dementia and oral health among subjects aged 75 years or older. *Gerodontology* **2012,** *29* (1), 36-42.

20. Tan, E. C. K.; Lexomboon, D.; Häbel, H.; Fastbom, J.; Eriksdotter, M.; Johnell, K.; Sandborgh-Englund, G., Xerogenic Medications as a Predictor for Dental Health Intervention in People with Dementia. *J Alzheimers Dis* **2020,** *75* (4), 1263-1271.

21. Tiisanoja, A.; Syrjälä, A. M.; Tertsonen, M.; Komulainen, K.; Pesonen, P.; Knuuttila, M.; Hartikainen, S.; Ylöstalo, P., Oral diseases and inflammatory burden and Alzheimer's disease among subjects aged 75 years or older. *Special care in dentistry : official publication of the American Association of Hospital Dentists, the Academy of Dentistry for the Handicapped, and the American Society for Geriatric Dentistry* **2019,** *39* (2), 158-165.

22. Warr; en, J. J. C., J. M.; Levy, S. M.; Blanco, V. L.; Ettinger, R. L., Oral health of persons with and without dementia attending a geriatric clinic. *Special care in dentistry : official publication of the American Association of Hospital Dentists, the Academy of Dentistry for the Handicapped, and the American Society for Geriatric Dentistry* **1997,** *17* (2), 47-53.

23. Yang, B.; Tao, B.; Yin, Q.; Chai, Z.; Xu, L.; Zhao, Q.; Wang, J., Associations Between Oral Health Status, Perceived Stress, and Neuropsychiatric Symptoms Among Community Individuals With Alzheimer's Disease: A Mediation Analysis. *Frontiers in aging neuroscience* **2021,** *13*, 801209.

24. Arrivé, E.; Letenneur, L.; Matharan, F.; Laporte, C.; Helmer, C.; Barberger-Gateau, P.; Miquel, J. L.; Dartigues, J. F., Oral health condition of French elderly and risk of dementia: a longitudinal cohort study. *Community dentistry and oral epidemiology* **2012,** *40* (3), 230-8.

25. Choi, S.; Kim, K.; Chang, J.; Kim, S. M.; Kim, S. J.; Cho, H. J.; Park, S. M., Association of Chronic Periodontitis on Alzheimer's Disease or Vascular Dementia. *Journal of the American Geriatrics Society* **2019,** *67* (6), 1234-1239.

**Supplementary** **Figure S2. Publication bias detected by funnel plot, Egger’s test and Begg’s test for the association between dentition defects and Alzheimer's disease.**


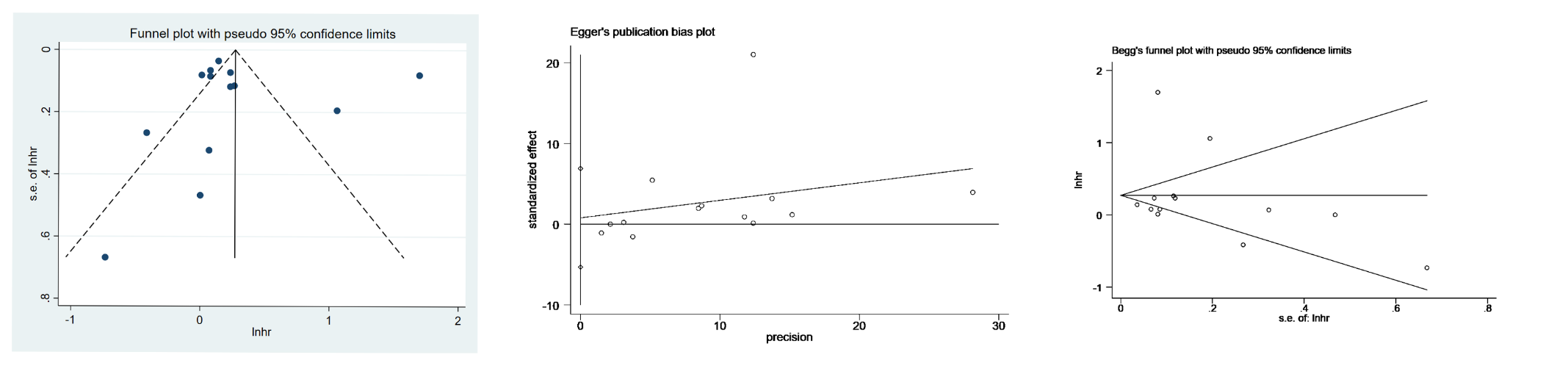


**Supplementary** **Figure S3. Sensitivity analysis of the association between dentition defects and Alzheimer's disease.**


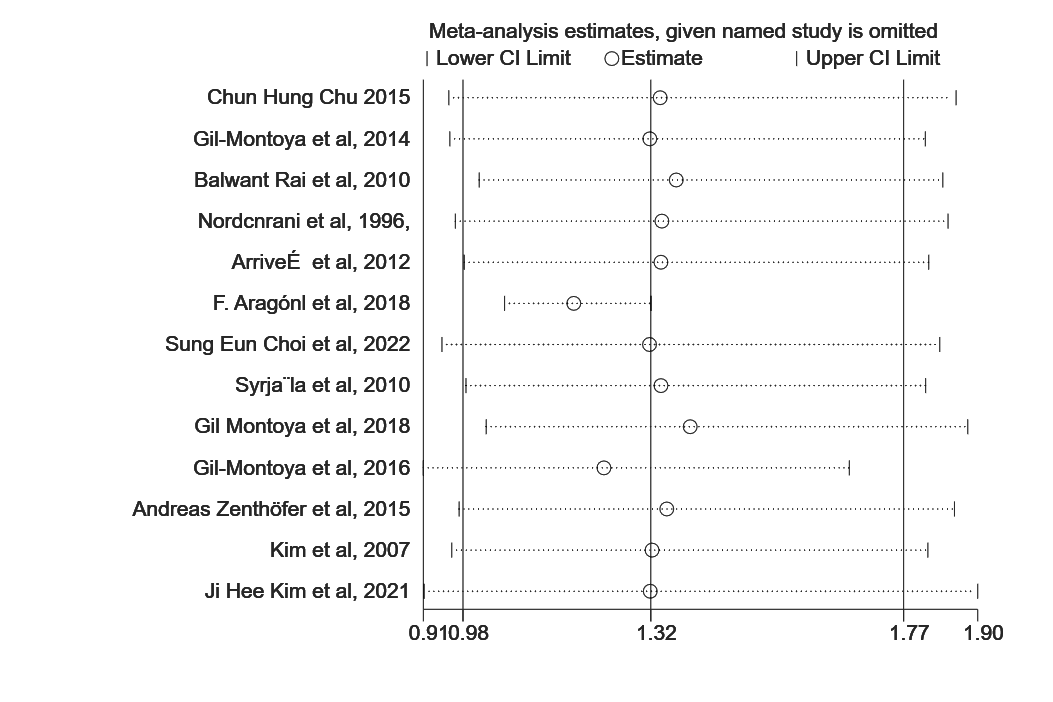


**Supplementary Table S4. Quality assessment of the included studies by Newcastle–Ottawa scale.**

| Author  (Publication Year) | Newcastle-Ottawa Scale | | | | | | | | | |
| --- | --- | --- | --- | --- | --- | --- | --- | --- | --- | --- |
|  | Selection | | | Comparability | | | Outcome | | | Total |
|  | a | b | c | d | e | f | g | h | i |  |
| Aragón 2018 | * | * | * |  |  | * | * |  | * | 6 |
| Arrive´ E 2012 | * | * | * |  |  | * | * | * | * | 7 |
| Balwant Rai 2010 | * | * | * |  | * | * | * |  | * | 8 |
| Sung Eun Choi 2022 | * | * | * |  | * | * | * |  | * | 8 |
| Chun Hung Chu 2015 | * | * | * |  |  |  | * |  | * | 5 |
| Anna-Maija 2012 | * | * | * |  | * | * | * |  | * | 7 |
| Jose´ A. Gil-Montoya 2014 | * | * | * |  | * | * | * |  | * | 7 |
| Jos e Antonio Gil-Montoya 2016 | * | * | * |  | * | * | * |  | * | 7 |
| Ji Hee Kim 2021 | * | * | * |  | * | * | * |  | * | 7 |
| Jae-Min Kim 2007 | * | * | * |  | * | * | * |  | * | 7 |
| Nordenram 1996 | * | * | * | * | * |  | * | * | * | 8 |
| Tha´ıs de Souza Rolim 2014 | * | * | * |  | * | * | * |  | * | 7 |
| John J. Warren 1997 | * | * | * |  | * | * | * |  | * | 7 |
| Andreas Zenthöfer 2015 | * | * | * |  | * | * | * |  | * | 7 |

1. Representativeness of the exposed cohort.
2. Selection of the non-exposed cohort.
3. Ascertainment of exposure.
4. Demonstration that outcome of interest was not present at start of study.
5. Comparability of cohorts on the basis of the design or analysis (adjusted for age).
6. Comparability of cohorts on the basis of the design or analysis (adjusted for any other factor).
7. Assessment of outcome.
8. Was follow-up long enough for outcomes to occur.
9. Adequacy of follow-up of cohorts.
